# Exploring knowledge and implementation gaps of activity-based therapy in centers lacking specialized spinal cord injury services: understanding therapists’ perspectives

**DOI:** 10.1038/s41394-024-00619-4

**Published:** 2024-03-21

**Authors:** Nicole Cesca, Chantal Lin, Zeina Abu-Jurji, Aaron Wexler, Jonas Mark, Shane McCullum, Rija Kamran, Brian Chan, Kristin E. Musselman

**Affiliations:** 1 Rehabilitation Sciences Institute, Temerty Faculty of Medicine, University of Toronto, Toronto, ON Canada; 2 KITE Research Institute, Toronto Rehabilitation Institute - University Health Network, Toronto, ON Canada; 3 Department of Physical Therapy, Temerty Faculty of Medicine, University of Toronto, Toronto, ON Canada; 4 Faculty of Health Sciences, Masters of Physiotherapy, McMaster University, Hamilton, ON Canada; 5 First Steps Wellness Centre, Winnipeg, MB Canada; 6 Stan Cassidy Centre for Rehabilitation, Horizon Health Network, Fredericton, NB Canada; 7 Rehabilitation Science, Faculty of Rehabilitation Medicine, University of Alberta, Edmonton, AB Canada; 8 Institute for Health Policy, Management and Evaluation, University of Toronto, Toronto, ON Canada

**Keywords:** Rehabilitation, Spinal cord diseases

## Abstract

**Study design:**

Qualitative exploratory

**Objectives:**

Rehabilitation following spinal cord injury (SCI) is a life-long process involving healthcare in a variety of settings, including facilities lacking SCI-specific services (i.e., non-SCI-specialized centers). Activity-based therapy (ABT) is a neurorestorative approach involving intensive, task-specific movement practice below the injury level. This study explored the existing knowledge, perceptions, and implementation of ABT among physical and occupational therapists working in non-SCI-specialized centers.

**Setting:**

Canadian hospitals and community clinics

**Design/methods:**

Semi-structured interviews were conducted with Canadian therapists who worked at non-SCI-specialized centers and treated at least one patient with SCI within the last 18 months. The Theoretical Domains Framework was used to develop interview questions that queried therapists’ experiences in delivering SCI rehabilitation, their understanding of ABT and experience with its implementation. Interviews were audio-recorded, transcribed verbatim and analyzed using interpretive description.

**Results:**

Four physical therapists and three occupational therapists, from diverse settings (i.e., acute care, inpatient rehabilitation, long-term care, outpatient rehabilitation, rural outpatient clinic) participated. Three themes were identified: (1) Available knowledge, resources and therapy time in non-SCI-specialized centers challenge ABT implementation, (2) How current therapy practices in non-SCI-specialized centers align with ABT and (3) Desire for ABT knowledge. Although participants were not familiar with the term ABT, it was identified that they were unknowingly incorporating some components of ABT into their practice. Participants expressed a keenness to learn more about ABT.

**Conclusion:**

Current knowledge and implementation of ABT in non-SCI-specialized centers is limited. Tailoring ABT education to therapists at non-SCI-specialized centers may increase ABT implementation.

## Introduction

Spinal cord injury or disease (SCI) is a life-altering condition that affects more than 85,000 Canadians [1]. The resulting sensory, motor, and autonomic dysfunction may cause a decrease in mobility and independence, along with an elevated susceptibility to secondary health conditions like cardiovascular dysfunction or musculoskeletal deterioration [2]. These consequences significantly affect the overall quality of life and societal engagement of individuals with SCI, indicating the need for effective therapy interventions across the continuum of care [3].

With the advancement in the understanding of neuroplasticity, the field of neurorehabilitation has experienced a shift from a compensatory approach to a restorative perspective [4]. Evidence suggests that task-specific, repetitive, and intensive neuromuscular activation below the level of injury, known as activity-based therapy (ABT), can promote neurological improvements and decrease the risk of secondary conditions associated with paralysis [5, 6]. This includes a reduction in cardiovascular and metabolic risk factors and improvements in bowel and bladder outcomes [5, 7]. For individuals with SCI, ABT encompasses various activities with the most common being treadmill training, muscle strengthening below the level of injury, overground walking, ergometer training, and load-bearing exercises [8, 9]. ABT sessions are distinguished by their high intensity and frequency, and inclusion of mass practice, task-specificity, sensory stimulation, and mental effort [8]. There are numerous benefits of ABT among the SCI population including improvements in mobility, neurological status, and quality of life [10, 11]. In Canada, SCI-specialized centers (i.e., centers with SCI-specific health services) have implemented ABT in various ways across the continuum of care [9, 12, 13], and an ABT Community of Practice (CoP) was formed to promote access to ABT after SCI [10].

Some Canadians living with SCI lack access to ABT due to their residential locations. Additionally, it is unknown if and how ABT is implemented in centers that lack SCI-specific services (i.e., non-SCI-specialized centers). SCI-specialized centers tend to be located in urban areas; however, 18% of the Canadian population lives in rural communities [14]. In comparison to urban areas, rural areas are limited in their access to certain health services and healthcare professionals, and as a result, often face poorer health outcomes [15]. Furthermore, individuals in these rural areas often travel outside of their local community to receive specialized services and many face financial strain, emotional burden, and family stress as a result [16].

The literature on healthcare accessibility for individuals with SCI residing in rural areas has focused on access to specialized physicians [17, 18], overlooking the critical role of other healthcare providers (e.g., rehabilitation professionals) and the services they provide (e.g., ABT or other forms of physical rehabilitation) [19, 20]. Understanding if and how ABT is delivered to individuals with SCI at non-SCI-specialized centers will contribute to the Canadian ABT CoP’s vision of improving the access and quality of ABT in Canada [10]. Hence, this study’s aim was to explore the existing knowledge, perceptions, and implementation of ABT among physical therapists (PTs) and occupational therapists (OTs) working in non-SCI-specialized centers.

## Methods

### Study design

This qualitative exploratory study was granted ethical approval by the Research Ethics Board of the University Health Network in Toronto, Canada. All participants provided written and verbal informed consent.

### Participant recruitment

PTs and OTs licensed to practice in Canada were eligible for the study. Inclusion criteria required that the participants worked at a non-SCI-specialized center but had experience working with at least one individual with SCI in the last 18 months. The target sample size of 7–10 participants was based on the concept of information power, which considers five study items (i.e., aim, specificity of the sample, use of theory, dialogue quality and analysis strategy) [21]. Three of the five items of information power suggested a smaller sample size of 7–10 participants was appropriate for this study (see Table 1). Moreover, this target sample size aligned with the sample sizes of similar research exploring the use of ABT in SCI-specialized centers [9, 12, 13]. Participants were recruited through snowball sampling, primarily utilizing the professional networks of members of the Canadian ABT CoP.

**Table 1:** Evaluation of sample size based on information power [21].

Items of information power	Study details	Favors small sample?
Study aim	- Research question is well-defined; however, participant’s experiences vary.	No
Sample specificity	- Specific sample of PTs and OTs, defined by clear inclusion criteria, yields a relatively homogeneous sample with reduced variation in identified themes.	Yes
Established theory	- Methodology for data collection incorporated a thorough review of existing literature on ABT and previous research exploring the use of ABT in SCI-specialized centers [9, 12, 13]. - Interview guide was shaped by the theoretical underpinnings of the Theoretical Domains Framework [22], ensuring a theory-rich perspective.	Yes
Quality of dialogue	- Interviews were conducted by an experienced qualitative interviewer with content expertize in both ABT and SCI, ensuring high-quality, informed dialogue relevant to the study’s focus.	Yes
Analysis strategy	- Cross-case analysis.	No

*PTs* physical therapists, *OTs* occupational therapists.

### Data collection

Semi-structured interviews were conducted with seven participants: four PTs and three OTs. All interviews were completed individually except one, which was conducted jointly with a PT and OT from the same facility. The interviews were conducted by phone from June 2022 to December 2022 and ranged in duration from 26 min to 45 min. All six interviews were conducted in English by a single researcher (KEM) to ensure rigor. The interview guide (Table 2) was modified from the guides used in prior qualitative studies exploring ABT use by therapists working in SCI-specialized centers [9, 12, 13]. The 14 domains of the Theoretical Domains Framework informed the development of questions that queried therapists’ knowledge and practice relating to SCI rehabilitation and ABT. The Theoretical Domains Framework was selected as it considers the factors that influence health behavior and decision-making [22]. At the beginning of the interview, participants were provided with a definition of ABT, specifically, they were told that ABT was “repetitive neuromuscular activation below the level of spinal injury, typically achieved through intensive, task-specific movement practice” [10].

**Table 2:** Interview guide.

Questions	Mapping to TDF domains [20] (for researcher reference only)
1. Please tell me about your past clinical experience working with clients with SCI. [Probes: Did you work with both complete and incomplete injuries? At what stage post-injury? How were the client(s) referred to your center? How long was this client(s) on your caseload?]	- Social/professional role and identity - Environmental context and resources
2. Please tell me about the rehabilitation for these individuals – what did it look like? [Probes: Therapeutic goals? Frequency of sessions? Activities performed during sessions? Role of assistants?]	- Knowledge - Social/professional role and identity - Goals
3. Do you and your colleagues use ABT at your site? [Probes: How would you describe your knowledge of ABT? For what therapeutic goals have you used ABT? Please tell me more about what the ABT sessions looked like. Was any equipment or technology used in these sessions, and if yes, how was it used?]	- Environmental context and resources - Goals - Knowledge - Attention, memory and decision processes
4. [If participant is using ABT] – What things have helped you use ABT in your practice?	- Environmental context and resources - Knowledge
5. [If participant is using ABT] – Have you experienced any challenges when trying to access and/or implement ABT in your practice? [Probe: Any challenge accessing or using equipment/technology for ABT?]	- Environmental context and resources - Knowledge - Skills - Social/professional role and identity - Beliefs about capabilities - Optimism - Beliefs about consequences - Reinforcement - Social influences - Emotion - Behavioral regulation
6. [If participant is NOT using ABT] – Do you feel like you would be able to incorporate ABT into your clinical practice? Why or why not? [Probes: Have you experienced any challenges when trying to access and/or implement ABT in your practice? Any challenge accessing or using equipment/technology for ABT?]	- Environmental context and resources - Knowledge - Skills - Social/professional role and identity - Beliefs about capabilities - Optimism - Beliefs about consequences - Reinforcement - Social influences - Emotion - Behavioral regulation
7. Do your clients have access to ABT and/or technologies that facilitate ABT in the community, after discharge from your center? [Probes: Are you aware of your clients wishing to access ABT and/or technologies that facilitate ABT in the community? And if yes, what was their experience?]	- Environmental context and resources - Knowledge - Optimism - Social influences
8. Would you like to increase your use of ABT and/or technology that facilitates ABT in your clinical practice? [Probe: If yes, in what way? If no, why?]	- Goals - Beliefs about capabilities - Beliefs about consequences - Optimism - Intentions - Behavioral regulation
9. Are you interested in learning more about ABT and how to implement ABT into clinical practice? Why or why not? If yes, how would you prefer to learn about ABT and how to implement ABT in clinical practice?	- Environmental context and resources - Knowledge - Social influences - Skills - Social/professional role and identity

*TDF* theoretical domains framework, *ABT* activity-based therapy, *SCI* spinal cord injury.

### Data analysis

An interpretive description approach was employed. This approach to analysis facilitated the identification of themes and patterns that are relevant for guiding clinical practice, as well as for further investigating health phenomena [23]. The analysis primarily involved three researchers (NC, CL, KEM), all of whom have physical therapy backgrounds; none work at non-SCI-specialized centers. Two researchers (NC and CL) independently immersed themselves in the data by reviewing all the transcripts and highlighting key points of interest. The first two transcripts were independently coded by the same two researchers, and initial codes were discussed, revised, and used to determine a preliminary codebook. Next, NC and CL independently coded the third interview, discussed the codes, and jointly refined the codebook, which was used to code the remaining three interviews. An iterative approach was employed, and NC, CL and KEM initially reflected on the relationships among the final codes and determined preliminary themes and subthemes. A fourth team member (JM), an experienced kinesiologist in ABT, reviewed preliminary themes, subthemes, and participant quotes and provided feedback, which was incorporated into the analysis. The results were then shared with the remaining team members, one of whom was a PT student (ZAJ) who had completed an internship in a rural and remote environment. A consensus among all team members was reached for the final themes and subthemes. NVivo (version 12, QSR International, Burlington, Massachusetts, USA) was used for data management.

## Results

Participants were from Ontario (*n* = 3) and Alberta (*n* = 4) and worked in healthcare centers across the care continuum. These settings included: acute care, which provides short-term, immediate medical treatment (*n* = 2); inpatient rehabilitation, which offers intensive therapy post-acute illness or injury with an extended stay in a specialized facility (*n* = 1); long-term care, a residential environment for ongoing medical and personal care for those unable to live independently due to chronic conditions (*n* = 1); outpatient rehabilitation, where individuals receive therapy without hospital admission, enabling them to return home post-treatment (*n* = 2); and a rural outpatient clinic, which provides outpatient rehabilitation services in less populated areas (*n* = 1) (Table 3).

**Table 3:** Participant demographics.

Participant	Site	Location	Profession	Years worked as a PT/OT in current health setting	Health setting
P01	1	Ontario	PT	10 years	Hospital: acute care
P02	2	Alberta	OT	6 months	Hospital: long-term care
P03	3	Alberta	PT	18 years	Hospital: acute care
P04	4	Ontario	PT	14 years	Hospital: outpatient neuro rehab
P05	4	Ontario	OT	9 years	Hospital: outpatient neuro rehab
P06	5	Alberta	OT	22 years	Hospital: inpatient neuro rehab
P07	6	Ontario	PT	3 years	Outpatient clinic in rural northern environment

*PT* physical therapist, *OT* occupational therapist.

Participants treated individuals with SCI at their non-SCI-specialized centers because of limited vacancy at specialized centers, geographic proximity, and convenience. One participant highlighted that their patient “actually came from a spinal center outside the city [because they] wanted to come back home here” (P01). Participants emphasized commuting long distances for specialized care can be challenging, especially for individuals with limited mobility and few transportation options. Furthermore, participants noted a rise in non-traumatic SCI (NT-SCI) cases at their non-SCI-specialized centers. One participant highlighted that individuals with NT-SCI were more commonly being referred to their non-specialized center instead of SCI-specialized facilities.

Three themes were identified that reflected participants’ experiences in delivering SCI rehabilitation in non-SCI-specialized centers: (1) Available knowledge, resources and therapy time in non-SCI-specialized centers challenge ABT implementation, (2) How current therapy practices in non-SCI-specialized centers align with ABT, and (3) Desire for ABT knowledge. Each theme encompassed 2–3 subthemes (Table 4).

**Table 4:** Themes and sub-themes.

Theme	Sub-theme
1. Available knowledge, resources and therapy time in non-SCI-specialized centers challenge ABT implementation	(a) Gaps in specialized knowledge related to SCI and ABT (b) Lack of access to resources for SCI rehabilitation and ABT implementation (c) Limited time for therapy and ABT delivery
2. How current therapy practices in non-SCI-specialized centers align with ABT	(a) Unconscious incorporation of some ABT principles into therapy at non-SCI-specialized centers (b) Focus on function and independence at non-SCI-specialized centers
3. Desire for ABT knowledge	(a) Interest in learning more about ABT (b) Accessible and tailored ABT education

### Theme 1: Available knowledge, resources and therapy time in non-SCI-specialized centers challenge ABT implementation

Prior to participating in the current study, none of the participants were familiar with the term ABT stating that “[ABT] is a newer term to me” (P06) and “I didn’t know much about [ABT]” (P07). However, after discussing the definition of ABT, participants realized they were familiar with the concept, with some drawing parallels to other therapy approaches stating, “Like I haven’t heard of [ABT], but … I was thinking it’s probably like the same as the constraint-induced movement therapy for stroke clients” (P04). During the discussion, participants commented on the challenges they encounter when applying ABT principles to the rehabilitation of individuals with SCI in a non-SCI-specialized center. Specifically, they identified: (a) gaps in specialized knowledge related to SCI and ABT, (b) lack of access to resources for SCI rehabilitation and ABT implementation, and (c) limited time for therapy and ABT delivery.

#### Subtheme 1a: Gaps in specialized knowledge related to SCI and ABT

Several gaps in knowledge were identified by participants, particularly in relation to familiarity with SCI, ABT and the specific resources required to effectively support this population. Many participants expressed a limited understanding of SCI, indicating a need for more comprehensive and specialized knowledge. Limited experience and knowledge were identified as factors contributing to discomfort: “The majority of the staff probably don’t feel comfortable working with [SCI] just based on their maybe limited experience or limited knowledge” (P06). The participants emphasized that specialized knowledge, such as ABT, which might not always be present outside of specialized centers, is needed when working with SCI: “I just find not being in the specialized center, like the competency is not always there when new things pop up and to be able to provide safe patient care, it’s like a lot of work” (P01). Additionally, participants mentioned that infrequent application of SCI-specific knowledge in daily practice often results in the need to “brush up on some old notes or some info that’s gone fuzzy over time … cause you don’t apply it every day” (P07). Lack of familiarity with specific devices and equipment needed for the SCI population and to assist with ABT implementation also posed a challenge for participants, “delay[ing] mobilizing and getting [patients] to move” (P01).

#### Subtheme 1b: Lack of access to resources for SCI rehabilitation and ABT implementation

The participants further attributed the challenges they face when delivering therapy to patients with SCI in non-SCI-specialized centers to a lack of access to resources, especially equipment. Participants acknowledged that they “don’t always have all of the resources, especially like physical equipment” (P05) that would be beneficial for ABT delivery. Participants recognized the need for specific equipment for therapy and ABT implementation, which their centers did not have: “Spinal cord injury is a relatively equipment-heavy diagnosis … it hasn’t always been the priority for us to have that stuff, so we’re often borrowing … equipment” (P05). Other types of resources were also highlighted as lacking in non-SCI-specialized centers. For example, one participant emphasized that, “We don’t necessarily have the same community resources or education that you would at the [specialized rehabilitation hospitals]” (P01).

#### Subtheme 1c: Limited time for therapy and ABT delivery

Participants identified that “time is definitely a barrier” (P02) when it comes to delivering intensive therapies like ABT. One participant acknowledged that it was often the therapist’s time constraints limiting use of intensive therapies like ABT rather than patient tolerance, “Some patients, I only have this much time to provide [therapy]. I guess they can do more, but this is all I can do” (P01). The limited amount of time available for therapists to dedicate to each patient was attributed to the high caseloads in non-specialized centers: “I always think of [my large] caseload and just like how much time we actually have to dedicate to each person” (P02). Furthermore, incorporating the needed equipment for individuals with SCI to help with ABT delivery, which participants were sometimes unfamiliar with, also consumed therapy time: “We do have a plinth but it’s very time-consuming cause we had to hoyer him into a chair” (P01). Finally, one participant expressed that the repetition of movement required for ABT following SCI, “is very challenging to do, [as it’s] very dependent a lot on timing and that doesn’t exist” (P05).

### Theme 2: How current therapy practices in non-SCI-specialized centers align with ABT

Participants commented on current therapy techniques they use with individuals with SCI in non-SCI-specialized centers. They spoke about: (a) unconscious incorporation of some ABT principles into therapy at non-SCI-specialized centers, and (b) focus on function and independence at non-SCI-specialized centers.

#### Subtheme 2a: Unconscious incorporation of some ABT principles into therapy at non-SCI-specialized centers

Despite all participants expressing limited familiarity with the term ABT, several acknowledged that they unconsciously incorporated certain ABT components such as weight-bearing exercises and exercises that promote neurological recovery into their current therapy practices: “I would say for the most part … I’m doing [ABT] kind of unconsciously … there would be a goal of neuro recovery [in therapy]” (P05). Another participant shared their adoption of ABT-related activities, like “weight bear[ing]” exercises, aimed at “helping the patient recover” however, noted they wouldn’t typically “care to call it ABT” (P01). This participant highlighted that while therapists may incorporate activities resembling ABT in their practice, they may not specifically identify or associate them with ABT itself: “We would kind of do those types of things, but … I don’t consider or think of it as [ABT]” (P01).

#### Subtheme 2b: Focus on function and independence at non-SCI-specialized centers

Participants highlighted that typically within non-SCI-specialized centers there is an emphasis on practicing functional tasks, but not at an intensity that would be considered ABT. For example, one participant stressed the focus on “gross functional movement and not doing repetitive type movements” (P01). Participants consistently prioritized and “tend[ed] to be more focused on functional activities” (P04). Additionally, participants emphasized “practicing dressing strategies” (P05) and “us[ing] the bathroom just to practice the transfers to the toilet” (P04). The main goal of therapy for many participants was to facilitate independence in tasks such as transfers and toileting. Treatment plans also typically included ADLs like “washing, grooming, dressing” (P06) and even “some iADLs [instrumental activities of daily living] in terms of kitchen practice” (P06). The approach to therapy primarily “focused on what [patients’] strengths [were] and being able to incorporate those into working towards some greater participation and independence with ADLs” (P06).

### Theme 3: Desire for ABT knowledge

Participants emphasized their desire for ABT knowledge by highlighting their (a) interest in learning more about ABT, as well as identifying the need for (b) accessible and tailored ABT education.

#### Subtheme 3a: Interest in learning more about ABT

Participants expressed a keen interest in learning more about ABT. Participants acknowledged that while they may incorporate certain aspects of ABT into their practice, they were eager to expand their knowledge and explore additional approaches: “Yeah, I wouldn’t mind [learning more about ABT]. I feel like I’m doing [ABT] but like what else could I be doing” (P01)? The sentiment of wanting to enhance their skill set was further expressed by a participant who mentioned that learning more about ABT is a “useful way to get a couple more tools in the tool bag” (P07).

#### Subtheme 3b: Accessible and tailored ABT education

Participants suggested preferred methods for receiving ABT education, such as “online modules” (P06) and “virtual learning” (P02). One participant highlighted their infrequent exposure to individuals with SCI and emphasized the importance of having “an accessible knowledge base, like online learning that, a therapist can just hop on at any time” (P06). Finally, the need to tailor education and resources to a therapist’s specific non-SCI-specialized care setting was highlighted by participants. One participant expressed aligning ABT educational principles with individual practices and emphasized educating on how “[ABT] relates to practice… in acute care, [as] it might be tailored differently” (P01).

## Discussion

The study findings offer insights into the challenges encountered by PTs and OTs in a variety of non-SCI-specialized settings, current therapy practices used in these environments, and therapists’ desire for ABT knowledge. Notably, the study highlighted therapists’ unconscious incorporation of certain ABT components into their existing therapy practices, despite their unfamiliarity with the term ABT. The findings shed light on the importance of addressing gaps in knowledge, lack of access to resources, and time constraints, which collectively impact the implementation of ABT in these non-SCI-specialized settings.

It was not surprising that the current participants were less familiar with ABT in comparison to clinicians working at SCI-specialized centers [9, 12, 13]. Yet, participants indicated they inadvertently incorporated components of ABT (e.g., task-specific movements, neuromuscular activation below the level of injury) into their practice. ABT is based on well-established principles of neuroplasticity [24], and most participants would have been exposed to these principles during their entry-to-practice education. This pre-existing familiarity with neuroplasticity principles may be an effective starting point for future ABT-related education and implementation efforts at non-SCI-specialized centers. Therapists’ familiarity with neuroplasticity principles may provide the psychological capability, or the mental strength and knowledge, to gain additional skills in ABT [25]. Participants indicated a desire to learn more about SCI and ABT through online education tailored to their care setting. Tailored education is known to play a pivotal role in facilitating behavioral change [26]. Therefore, providing therapists at non-SCI-specialized centers with SCI and ABT educational resources that are designed for their environment is needed.

Participants encountered patients with SCI in their non-SCI-specialized center due to close geographical proximity between the center and patient’s home. The ability to reach healthcare is a key dimension of access to specialized services according to Lesveque and colleagues [27]. This dimension includes the ability to acquire the requisite transportation and social support to reach qualified professionals, equipment, and other resources [27]. Long distances between patients’ homes and community-based ABT clinics were previously identified as a barrier to accessing ABT for community-dwelling Canadians living with chronic SCI [13]. To address this barrier, we suggest increasing the number of centers with therapists knowledgeable about SCI and ABT, as well as expanding the mode of ABT delivery (e.g., virtual services). The COVID-19 pandemic prompted an uptake in virtual rehabilitation and remote care [28], creating an opportunity for specialized services to transcend geographical barriers and reach therapists and patients in remote or underserved areas [29, 30]. Prior work in rural areas has shown the effectiveness of implementing telehealth initiatives to enhance access to specialized emergency medical care [31]. Applying a similar strategy to the field of rehabilitation has the potential to facilitate stronger partnerships and better communication between non-specialized and specialized centers and extend the reach of specialized services, such as ABT.

It was also noted that individuals with NT-SCI were increasingly seen at non-SCI-specialized centers in Canada. Yet, functional outcomes of individuals with NT-SCI are greater when their rehabilitation occurs in a specialized center rather than a non-SCI-specialized setting [32]. Interestingly, in Australia, individuals with NT-SCI were less likely to be admitted to a specialized rehabilitation unit following injury in comparison to those with traumatic SCI [32]. As individuals with NT-SCI are more likely to present with incomplete spinal damage [31], their higher level of function may be why they are not directed toward specialized services. In Canada and other developed countries, there is a rising incidence of NT-SCI due to aging populations [31, 33–35]. With this shift in SCI demographics, the importance of providing high-quality, SCI-specific rehabilitation in non-SCI-specialized centers is evident, as these centers are increasingly likely to encounter individuals with SCI [35].

One study limitation was that all interviews were conducted by phone, preventing capture of non-verbal cues that could have added valuable information to the findings [36]. Additionally, only two Canadian provinces and territories were represented.

In the future, the findings from this study may be formally synthesized with previous investigations examining clinicians’ perspectives on ABT implementation in SCI-specialized centers [9, 12, 13] to understand common challenges and facilitators for ABT implementation in Canada. The resulting synthesis may help identify effective implementation strategies for ABT in the Canadian healthcare context. Another potential future direction of the current work is the development and dissemination of educational resources on SCI and ABT for clinicians working at non-SCI-specialized centers, along with an evaluation of their impact on clinician knowledge and use of ABT.

## Conclusion

This study offered insights into therapists’ experiences with SCI rehabilitation and ABT at non-SCI-specialized centers. It was found that knowledge and implementation of ABT in non-SCI-specialized centers were limited. The findings highlighted the challenges that therapists in these centers experience, such as lacking knowledge, resources and time for ABT and SCI rehabilitation more broadly. Although therapists at non-SCI specialized centers were unfamiliar with the term ABT, many of them unconsciously incorporated components of ABT into their therapy practices. Furthermore, they expressed a desire for ABT education tailored to the needs of non-SCI-specialized settings. Altogether the findings may be used to develop targeted educational programs and implementation strategies to increase ABT access in non-SCI-specialized settings.

## Data Availability

The datasets generated during the current study are not publicly available as the interview transcripts may contain information that could compromise the privacy of research participants. The datasets are available from the corresponding author on reasonable request and approval from the research ethics board.
